# TFRC, associated with hypoxia and immune, is a prognostic factor and potential therapeutic target for bladder cancer

**DOI:** 10.1186/s40001-024-01688-9

**Published:** 2024-02-09

**Authors:** Runhua Tang, Haoran Wang, Jianyong Liu, Liuqi Song, Huimin Hou, Ming Liu, Jianye Wang, Jianlong Wang

**Affiliations:** 1 Department of Urology, Beijing Hospital, National Center of Gerontology, Institute of Geriatric Medicine, Chinese Academy of Medical Sciences, Beijing, People’s Republic of China; 2 Graduate School of Peking Union Medical College, Chinese Academy of Medical Sciences, 9 DongDan Santiao, Beijing, 100730 China; 3 Fifth School of Clinical Medicine, Peking University, Beijing, China

**Keywords:** Hypoxia, Immune, Immune microenvironment, Drug sensitivity, BLCA, TFRC

## Abstract

**Background:**

Bladder cancer is a common malignancy of the urinary system, and the survival rate and recurrence rate of patients with muscular aggressive (MIBC) bladder cancer are not ideal. Hypoxia is a pathological process in which cells acquire special characteristics to adapt to anoxic environment, which can directly affect the proliferation, invasion and immune response of bladder cancer cells. Understanding the exact effects of hypoxia and immune-related genes in BLCA is helpful for early assessment of the prognosis of BLCA. However, the prognostic model of BLCA based on hypoxia and immune-related genes has not been reported.

**Purpose:**

Hypoxia and immune cell have important role in the prognosis of bladder cancer (BLCA). The aim of this study was to investigate whether hypoxia and immune related genes could be a novel tools to predict the overall survival and immunotherapy of BLCA patients.

**Methods:**

First, we downloaded transcriptomic data and clinical information of BLCA patients from The Cancer Genome Atlas (TCGA) and Gene Expression Omnibus (GEO) databases. A combined hypoxia and immune signature was then constructed on the basis of the training cohort via least absolute shrinkage and selection operator (LASSO) analysis and validated in test cohort. Afterwards, Kaplan–Meier curves, univariate and multivariate Cox and subgroup analysis were employed to assess the accuracy of our signature. Immune cell infiltration, checkpoint and the Tumor Immune Dysfunction and Exclusion (TIDE) algorithm were used to investigate the immune environment and immunotherapy of BLCA patients. Furthermore, we confirmed the role of TFRC in bladder cancer cell lines T24 and UMUC-3 through cell experiments.

**Results:**

A combined hypoxia and immune signature containing 8 genes were successfully established. High-risk group in both training and test cohorts had significantly poorer OS than low-risk group. Univariate and multivariate Cox analysis indicated our signature could be regarded as an independent prognostic factor. Different checkpoint was differently expressed between two groups, including CTLA4, HAVCR2, LAG3, PD-L1 and PDCD1. TIDE analysis indicated high-risk patients had poor response to immunotherapy and easier to have immune escape. The drug sensitivity analysis showed that high-risk group patients were more potentially sensitive to many drugs. Meanwhile, TFRC could inhibit the proliferation and invasion ability of T24 and UMUC-3 cells.

**Conclusion:**

A combined hypoxia and immune-related gene could be a novel predictive model for OS and immunotherapy estimation of BLCA patients and TFRC could be used as a potential therapeutic target in the future.

**Supplementary Information:**

The online version contains supplementary material available at 10.1186/s40001-024-01688-9.

## Introduction

Bladder cancer (BLCA) is a common malignant cancer in urinary system [1]. Unlike other cancers, BLCA can be divided into non-muscle-invasive (NMIBC) and muscle-invasive (MIBC) bladder cancer [2]. Non-invasive and invasive tumors differ in clinical presentation, tumor invasiveness, pathological type and prognosis [3]. Besides, muscle-invasive cancers are more likely to have recurrence. As for treatment, considering approximately 75% of patients with BC present with a disease confined to the mucosa (stage Ta, CIS) or submucosa (stage T1) [4], the standard care strategy includes a combination of endoscopic resection and intravesical bacillus of Calmette-Guerin (BCG) instillation, particularly for patients who have high-risk NMIBC [5]. While it is well established that BCG immunotherapy is currently the best treatment for NMIBC [6], up to 40% of patients show no response to this treatment. A study found that multifocality, lymphovascular invasion and high grade on re-TURB were independent predictors for response to BCG treatment [7]. For MIBC patients, radical surgery is generally selected, and adjuvant therapy such as chemotherapy and immunotherapy is decided according to postoperative pathological conditions [8, 9]. However, even after comprehensive treatment, the survival rate and recurrence rate of MIBC patients are still not ideal [10]. Therefore, predictors of early prognosis and immunotherapy responsiveness of bladder cancer need to be identified.

Hypoxia is a pathological process by which cells acquire specific properties to adapt to a hypoxic environment [11]. Hypoxia can affect the proliferation, invasion and immune response of bladder cancer cells [12]. This is believed to be associated with changes in the tumor microenvironment (TME) [13, 14]. Nevertheless, hypoxia can also influence the radiotherapy, chemotherapy and even the genetic instability and malignant progression of MIBC [15, 16]. These studies indicated that hypoxia is closely related to the prognosis of bladder cancer and thus can be thought to be a potential treatment.

TME is a complex system composed of cancer cells, extracellular matrix, immune cells and other molecules, which plays an important role in the occurrence and development of bladder cancer [17–19]. It is thought that the loss of immune cell function in the tumor microenvironment can free malignant tumors from immune surveillance [20]. In recent years, immune-related genes have attracted more and more attention from researchers. Xu et al. found immune-related genes (IRGs) were associated with prognosis in RCC [17]. Xiao et al. found IRGs were involved in T-cell activation, cell killing, and NK cell activity and other biological processes and were strongly related to OS [22].

Up to now, the relationship between the expression of hypoxia- and immune-related genes and BLCA has not been studied in detail. In this study, a risk score model based on eight hypoxia- and immune-related genes was constructed and validated by GEO database. The AUC values and survival analysis results illustrated the feasibility and accuracy of the risk score model. TIDE and TCIA database showed the high value of risk score model in immunotherapy response. Taking together, our results demonstrated the high value of risk score model and nomogram for the prediction of survival and immunotherapy for patients with BLCA.

## Methods

### Data collection

The gene expression data and corresponding clinicopathological characteristics (including age, gender, survival status, survival time, tumor grade, tumor stage, TNM stage.) of bladder cancer patients were downloaded from The Cancer Genome Atlas (TCGA) database (https://gdc-portal.nci.nih.gov/), which including 412 BLCA samples and 19 normal bladder samples. GSE13507 from Gene Expression Omnibus (GEO) database (https://www.ncbi.nlm.nih.gov/geo/) was downloaded as test cohort. Simple nucleotide variation (SNV) data were obtained from TCGA database, and tumor mutation burden (TMB) was defined as the total number of nonsynonymous alterations (SNVS or indels) for each patient.

### Identification of differentially Expressed HRGs and IRGs

254 hypoxia-related genes were collected from GSEA database (http://www.gsea-msigdb.org/gsea) [23, 24]. IMMPORT database (https://www.immport.org/home) was used to collect IRGs and 1794 IRGs were selected. “limma” R package was employed to identify the differentially expressed genes (DEGs) between tumor and normal samples with FDR < 0.05 and |logFC| > 1. These DEGs were visualized by heatmap and volcano plot. Venn diagrams were used to graphically describe the combination, intersection, and difference between DEGs, HRGs and IRGs [25].

### Pathway enrichment analysis

To better understand the pathways and bioactivities of these intersection genes, Kyoto Encyclopedia of Genes and Genomes (KEGG) was employed to analysis signaling pathways and Gene Ontology (GO) was used to analyze biologic processes, molecular functions, and cellular components through “clusterProfiler” R package. These analyses were aimed to determine whether the genes filtered for further investigation were indeed involved in hypoxia and immunity.

### Construction of risk model based on intersection genes

First, we used univariate Cox regression analysis to identify the relationship between the expression of HRGs and IRGs and overall survival. Then the least absolute shrinkage and selection operator (LASSO) and multivariate Cox regression were used to determine the optimal prognostic factors of the model. After that, risk score model was calculated according to the following formula:

The Coef means the coefficient of each relevant mRNA in the risk model. Based on the median risk score, TCGA cohort and GEO cohort was divided into high-risk and low-risk groups while the TCGA cohort was defined as train cohort and GEO as test cohort.

### Analysis and validation of risk model

Kaplan–Meier curves were plotted to illustrate the different OS between high- and low-risk groups in train and test cohorts via “survival” and “survminer” R packages. Patients’ survival status and survival time were also estimated. Besides, progression-free survival (PFS) between subgroups of BLCA patients in test cohort was plotted via Kaplan–Meier curves. Univariate and multivariate Cox regression were conducted to assess whether risk model could be regarded as independent risk parameter. Principal component analysis (PCA) were employed to the model feasibility and accuracy. The accuracy, sensitivity, and specificity of the model were estimated by the Receiver Operating Characteristic (ROC) curves.

### Identification the association of risk score model and clinicopathological features

Heatmap was plotted by “pheatmap” R package to illustrate the relationship between eight genes’ expression and different clinicopathological features including risk, age, gender, stage, grade, T, N and M. Box plots illustrated the different risk score in different clinicopathological features.

### Construction of nomogram

We constructed a nomogram survival model via “rms” R package to predict the 1-, 3- and 5-year survival probability. Calibration curves, ROC curves, univariate Cox and multivariate Cox analysis were employed to illustrate the high accuracy of nomogram.

### Analysis of DEGs between subgroups

The different expressed genes (DEGs) between subgroups were identified with FDR < 0.05 and |log2FC| ≥ 1. Gene set variation analysis (GSVA) by “GSVA”R package was used to estimate the functional enrichment pathways in two subgroups.

### Estimation of the immune cell infiltration, immune microenvironment, and genetic alterations analysis

The immune microenvironment including stromal score (stromal content), immune score (degree of immune cell infiltration), ESTIMATE score (a composite marker of stroma and immunity) and tumor purity was illustrated by “estimate” R package. The immune cell infiltration between two groups were calculated by CIBERSORT and visualized in box plots. The coalition of different immune cell and OS were plotted through Kaplan–Meier curves. TMB was analyzed by the “maftools” R package. The survival probability of low-TMB and high-TMB patients in subgroups was also assessed.

### Checkpoint estimation and immune therapy estimation

Immune-checkpoint between high-and low-risk groups was estimated. The expression of common checkpoint including CTLA4, PD-L1, PDCD1, LAG3 and HAVCR2 between two subgroups was investigated, and their coalition with risk score was also searched. Furthermore, the tumor immune dysfunction and exclusion (TIDE) algorithm was conducted to determine response to immunotherapy in TCGA cohort. Then we used Cancer Immune Atlas (TCIA) database (http://tcia.at/) to obtain immunotherapy files of BLCA patients and further investigated the potential predictive roles of risk score in immunotherapy response in different treatments.

### Potential drugs prediction

Furthermore, we calculated the chemotherapy sensitivity of each BLCA patient according to the Genomics of Drug Sensitivity in Cancer (GDSC) database. IC50 values for each chemotherapy drug were further determined by regression analysis. IC50 values for each drug was further determined and was compared between high-risk and low-risk groups via boxplots.

### Cell lines and culture conditions

T24 and UMUC3 cells are acquired from Procell Life Science & Technology Co., Lt. T24 cells are cultured with RPMI-1640 medium supplemented with 10% FBS and 1% ABAM, while UMUC3 cells are cultured in the UM-UC-3 Cells Complete Medium. All cells were maintained at 37 ℃ in a humidified 5% CO_2_ incubator.

### Edu assay

T24 and UMUC3 cells were seeded at a density of 4000cells/well into 96-well plates then transported with two independent siRNA targeting TFRC. After 48 h, cells were added with EdU and continued incubating for another 2 h. Then, the cells were fixed with a 4% paraformaldehyde solution for 30 min. The staining process was conducted in accordance with the manufacturer's instructions. Images were captured under microscope, and the number of positive cells was quantified utilizing imageJ software.

### Wound healing assay

T24 and UMUC3 cells were seeded in 6-well plates in triplicates for each condition, two of which were treated with two independent si-TFRC. The cells were allowed to grow until full confluency was achieved. A 10 µL pipette was used to form a uniform size wound in the center of the cell monolayer for each condition, and a photograph was taken at 0 h. Cells were then observed for wound healing and photomicrograph at 48 h was taken under microscope.

### Invasion assay

The cells were transfected with either control or siRNA and seeded on the Matrigel-coated PET membrane in the upper compartment. The lower compartment was filled with complete growth media and the plates were maintained at 37 °C for 24 h. Cells that migrated to the lower side of membrane were fixed and stained with crystal violet. The cells were then photographed using a light microscope and the number of cells were counted using ImageJ software.

### Statistical analysis

All statistical analyses were used R software (Version 4.1.3). *p* < 0.05 was served as the cutoff criterion.

## Results

### Identification of differently expressed hypoxia-related genes and immune-related genes in BLCA patients

**Fig. 1:**
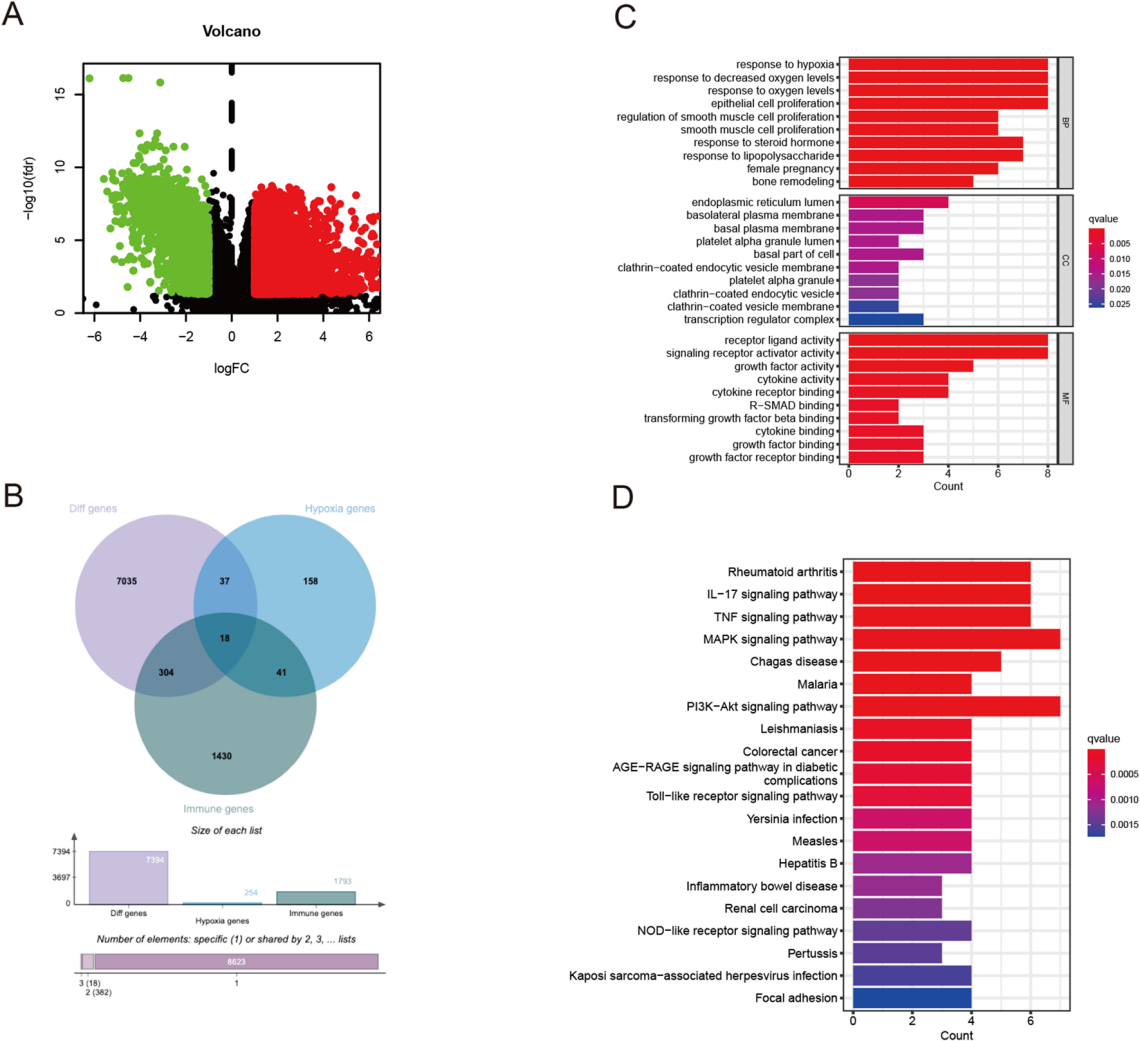
Identification and Functional Analysis of Hypoxia-Related Genes and Immune-Related Genes. **A** Volcano plot of differently expressed Hypoxia-Related Genes and Immune-Related Genes in BLCA patients. **B** Identification of intersection genes from DEGs, HRGs and IRGs. **C** The bar plot of GO enrichment analysis. **D** The bar plot for KEGG enrichment analysis

We screened 7394 differently expressed genes between normal and tumor tissue samples (Additional file 1: Fig. S1 and Additional file 3: Table S1), including 2848 downregulated genes and 4546 upregulated genes with FDR value < 0.05 and |logFC|> 1 (Fig. 1A). Using public databases, we identified 254 hypoxia-related genes (HRGs) and 1793 immune-related genes (IRGs). Then we employed Venn diagram to identify intersection genes from DEGs, HRGs and IRGs. Finally, 18 genes were filtered (Fig. 1B, Additional file 4: Table S2) and considered the differentially expressed hypoxia- and immune-related genes. These genes were used for subsequent analysis.

### Functional analysis of hypoxia-related genes and immune-related genes pathways

GO function analysis showed that these genes were involved in hypoxia, cell proliferation, steroid hormone and lipopolysaccharide (Fig. 1C). KEGG pathway analysis showed that these genes were involved in PI3K-Akt signaling pathway, MAPK signaling pathway, Rheumatoid arthritis, IL-17 signaling pathway and TNF signaling pathway (Fig. 1D). These results indicated these genes were closely related to hypoxia and immunity.

### Construction and validation of risk score model

**Fig. 2:**
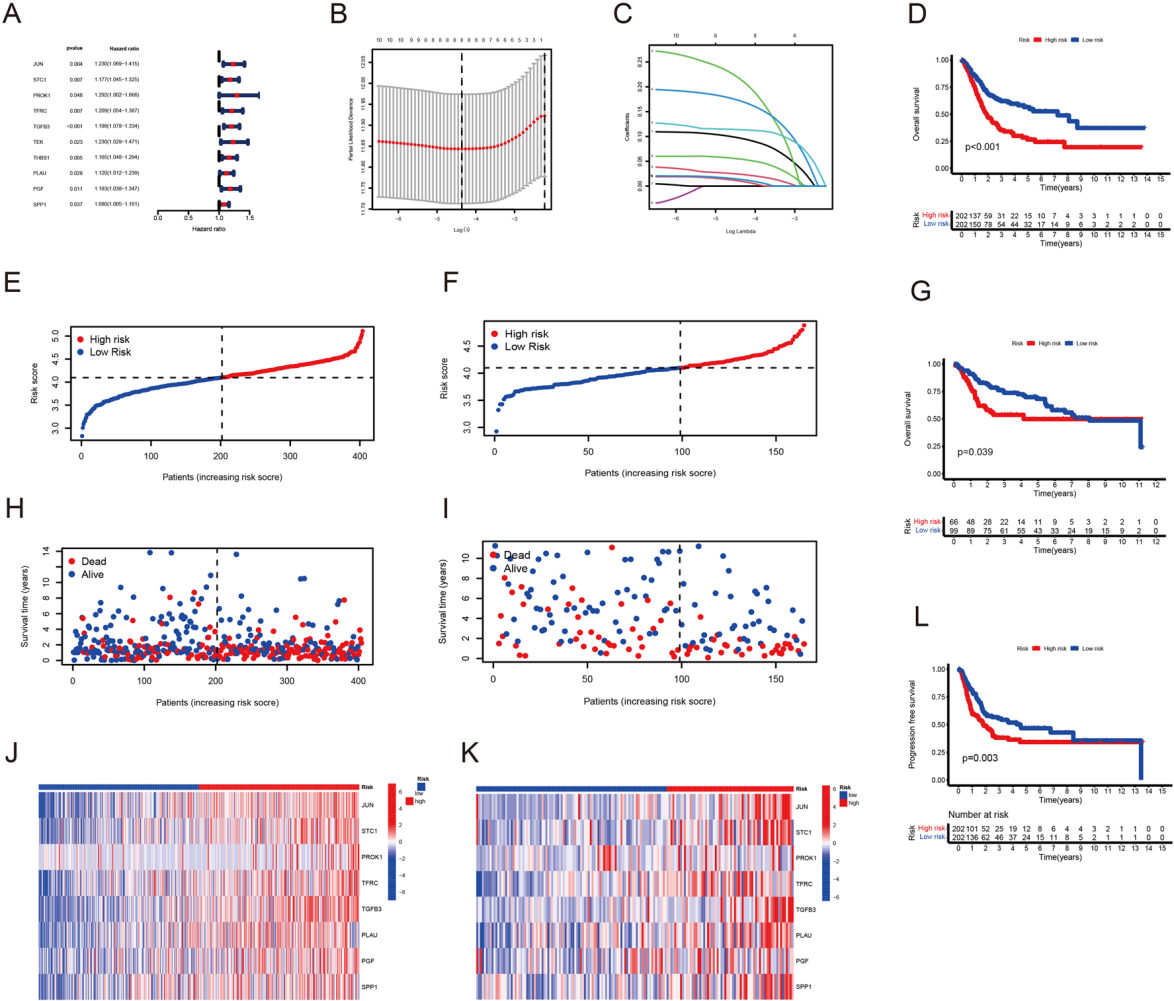
Correlation between the risk score model and overall survival of BLCA patients. **A** The relationship between intersection genes and overall survival. **B**, **C** LASSO regression analysis to optimize the risk score model for patients with BLCA. **E** The distribution of risk scores in the train cohort. **F** The distribution of risk scores in the test cohort. **H** The survival status of patients in the train cohort. **I** The survival status of patients in the test cohort. **J** Heat map of 8 genes expression in the train cohort. **K** Heat map of 8 genes expression in the test cohort. **D** Kaplan–Meier curves of survival in the train cohort. **G** Kaplan–Meier curves of survival in the test cohort. **L** Kaplan–Meier curves of PFS in the train cohort

**Fig. 3:**
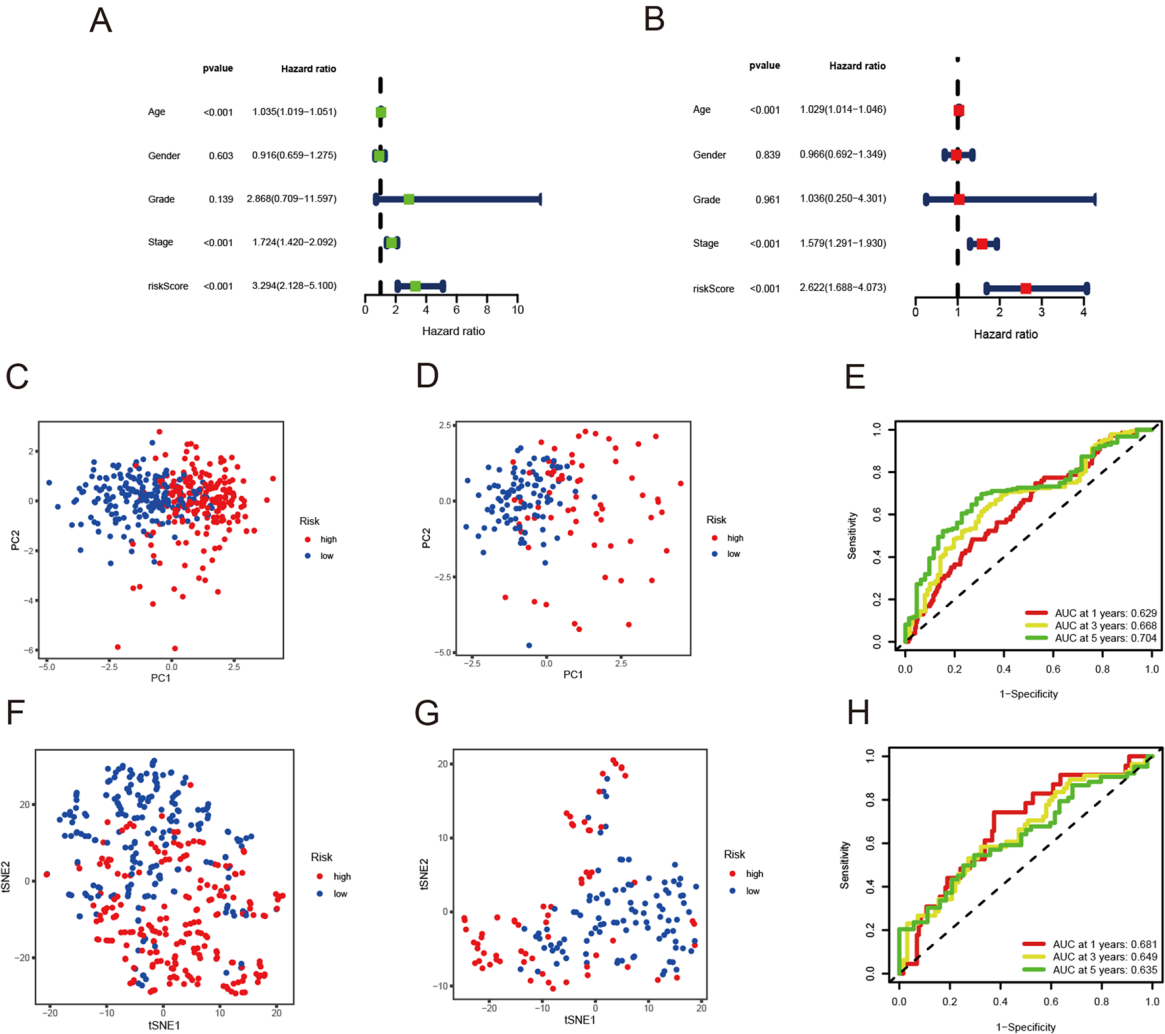
Validation of the risk score model. **A** Univariate cox proportional hazard model in the train cohort. **B** Multivariate cox proportional hazard model in the train cohort.** C** PCA analysis in the train cohort. **D** PCA analysis in the test cohort. **F** t-SNE analysis in the train cohort. **G** t-SNE analysis in the test cohort. **E** Time-dependent ROC curves of the risk score model for predicting 1-, 3- and 5-years in the train cohort. **H** Time-dependent ROC curves of the risk score model for predicting 1-, 3- and 5-years in the test cohort

First, we used univariate Cox analysis to identify the relationship between these genes and overall survival, and 10 genes were screened (Fig. 2A). To optimize model, we employed LASSO regression analysis (Fig. 2B, C). Therefore, a risk score model including JUN, STC1, PROK1, TFRC, TGFB3, PLAU, PGF and SPP1 was established (Additional file 5: Table S3). Subsequently, we divided BLCA patients in TCGA and GEO cohorts into low and high-risk groups according to the median risk score (Fig. 2E, F). The prognosis of BLCA patients in the low-risk group was better than that in the high-risk group (Fig. 2H, I). The expression levels of the 8 hypoxia- and immune-related genes were visualized in the heatmap (Fig. 2J, K). Kaplan–Meier curves illustrated that high-risk group had worse OS than low-risk group both in train cohort and test cohort (*p* < 0.001 and *p* = 0.039, respectively) (Fig. 2D, G). High-risk group was also observed worse PFS than low-risk group in train cohort (*p* = 0.003) (Fig. 2L). The univariate Cox and multivariate Cox analysis was performed in the train cohort, which indicated our risk score model was an independent prognostic factor (*p* < 0.001) (Fig. 3A, B). PCA and t-SNE analysis illustrated our model could distinguish dimensions among different groups both in train and test cohorts (Fig. 3C, D, F, G). ROC analysis showed the AUCs for 1-, 3- and 5-year survival was 0.629, 0.668 and 0.704, respectively in train cohort and 0.681, 0.649 and 0.635, respectively, in test cohort (Fig. 3E, F). These data suggested that our model could be helpful in predicting the outcome of BLCA patients.

### The coalition between different clinical features and risk model

**Fig. 4:**
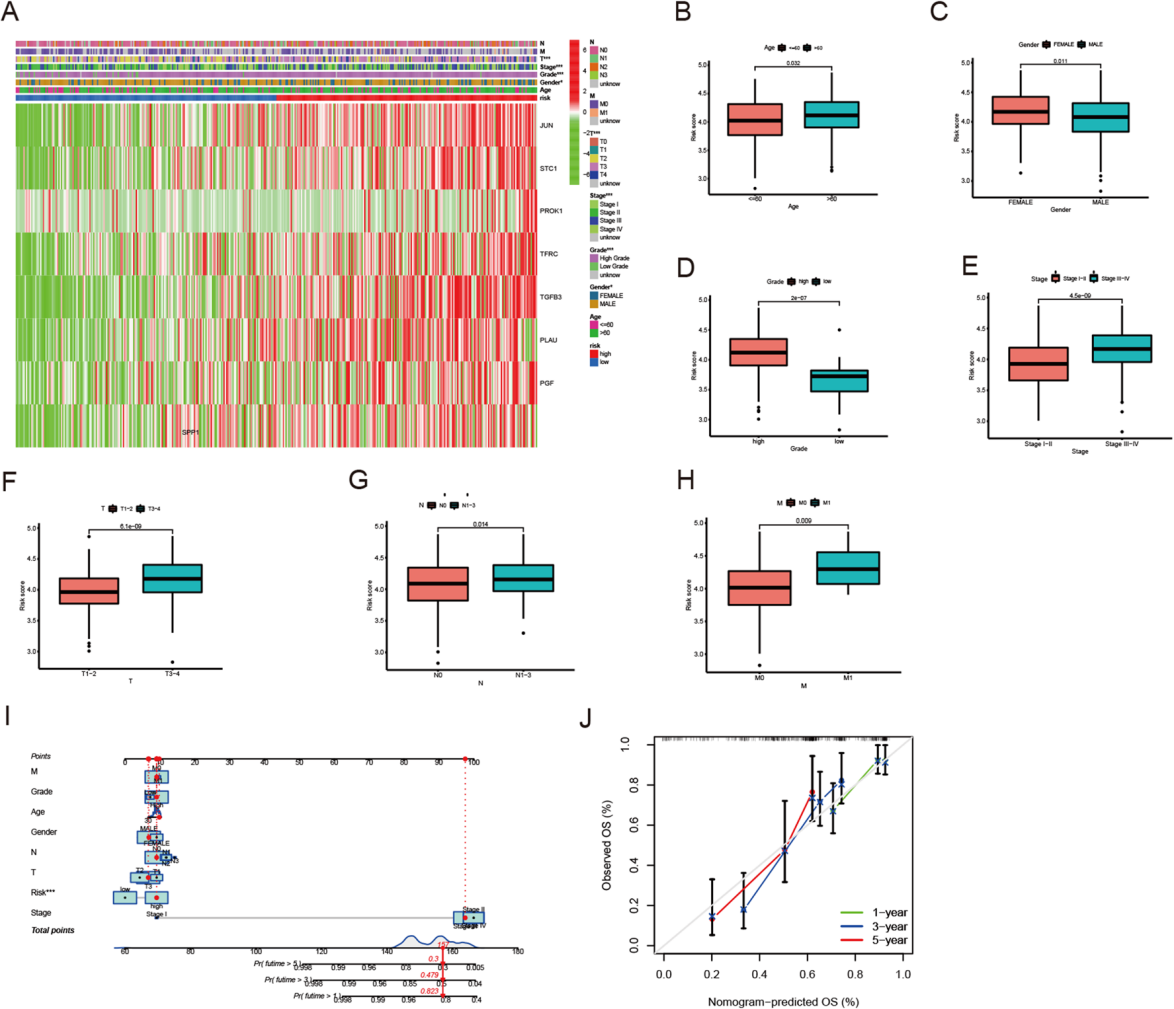
The clinical correlation analysis and predictive nomogram. **A** Clinical correlation analysis heatmap. **B–H** The relationship between eight genes’ expression and different clinicopathological features. **I** Nomogram to evaluate the survival risk of particular BLCA patients. **J** Calibration curves for nomogram-predicted OS at 1, 3 and 5 years

We conducted a heatmap to illustrate the coalition between different clinical features and risk model (Fig. 4A). Risk score has close relation with age (> 60 years or ≤ 60 years), gender (female or male), grade (high or low), stage (stage I–II or stage III–IV), T (T1–2 or T3–4), N (N0 or N1–3) and M (M0 or M1) (Fig. 4B–H). These results indicated our risk score model was in good agreement with different clinical and pathological features.

### Construction of predictive nomogram and calibration curves

Based on the previous results, we constructed a predictive nomogram to provide a more convenient and accurate tool that can predict the survival risk of particular BLCA patients (Fig. 4I). Our results showed that nomograms could be used as an effective tool for prognostic assessment of BLCA patients, and the calibration curves indicated our nomogram had high accuracy in BLCA patients’ survival rate (Fig. 4J).

### Functional enrichment analysis of DEGs between high- and low-risk groups

**Fig. 5:**
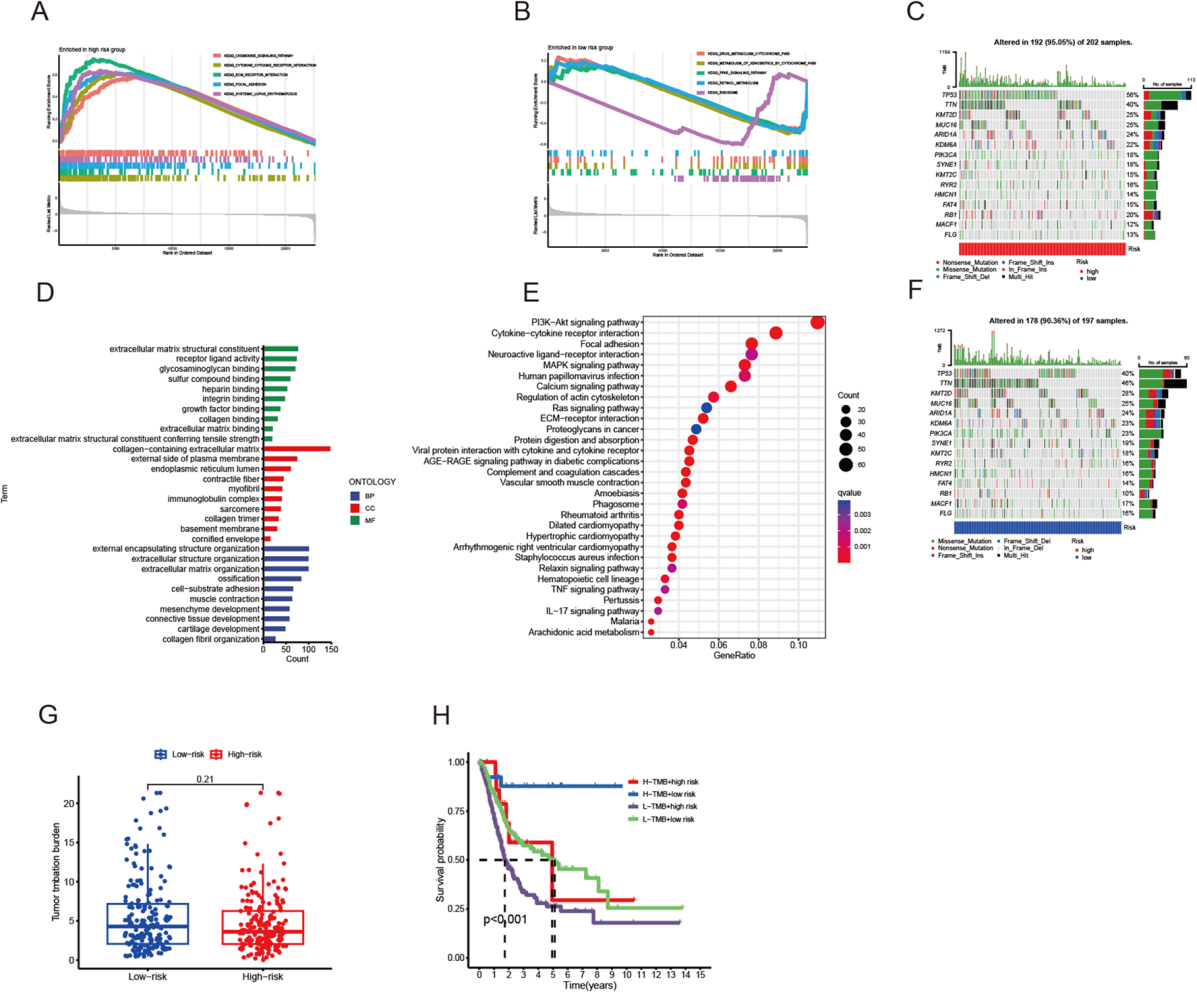
Functional enrichment and tumor mutation analysis between high- and low- risk groups. **A** GESA analysis in the high- risk group. **B** GESA analysis in the low- risk group. **D** The bar plot of GO enrichment analysis. **E** The bubble plot for KEGG enrichment analysis. **C**, **F** Differences in mutation frequency between high- and low- risk groups. **G** Tumor mutation burden between high- and low-risk groups. **H** Survival analysis between subgroups

With FDR value < 0.05 and |logFC|> 1, we identified 1547 differently expression genes between subgroups, including 248 downregulated genes and 1299 upregulated genes (Additional file 6: Table S4). Then we performed GSEA, GO and KEGG analysis to show the enriched pathways. GSEA indicated the chemokine signaling pathway, cytokine-cytokine receptor interaction, ECM receptor interaction, focal adhesion and systemic lupus erythematosus were enriched in high-risk group, indicating high- risk group might eager to have metastasis (Fig. 5A, B). The results of GO and KEGG enrichment analysis also illustrated that collagen-containing extracellular matrix and PI3K-Akt pathway was activated in high-risk group (Fig. 5D, E).

### Tumor mutation burden and survival analysis

The maftool package in R software is utilized to identify the top 15 mutation genes in high-risk and low-risk groups (Fig. 5C, F). Tumor mutation burden (TMB) between two groups had no significantly different (Fig. 5G). Nevertheless, subsequent analysis indicated that while combined with risk score, high TMB with low risk had better outcome and low TMB with high risk had worse outcome (Fig. 5H).

### Analysis of TME, immune cell infiltration and immune-checkpoint

**Fig. 6:**
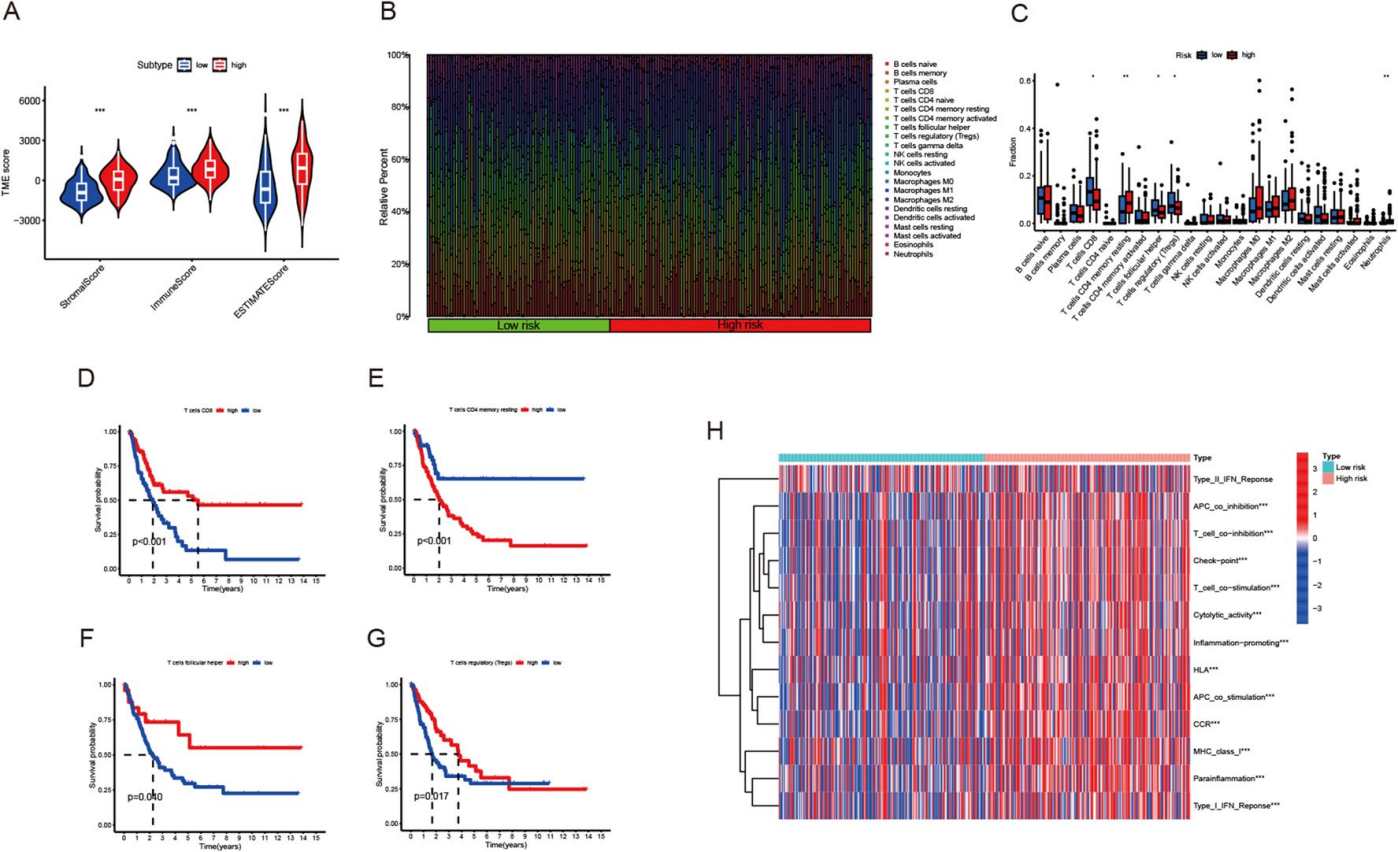
Correlation between tumor immune cell infiltration and risk score. **A** Correlation between TME and risk score. **B**, **C** The composition of subpopulations of immune cells in high- and low-risk groups. **D**–**G** Survival curves in different subtypes. **H** The different immune functions between two groups

**Fig. 7:**
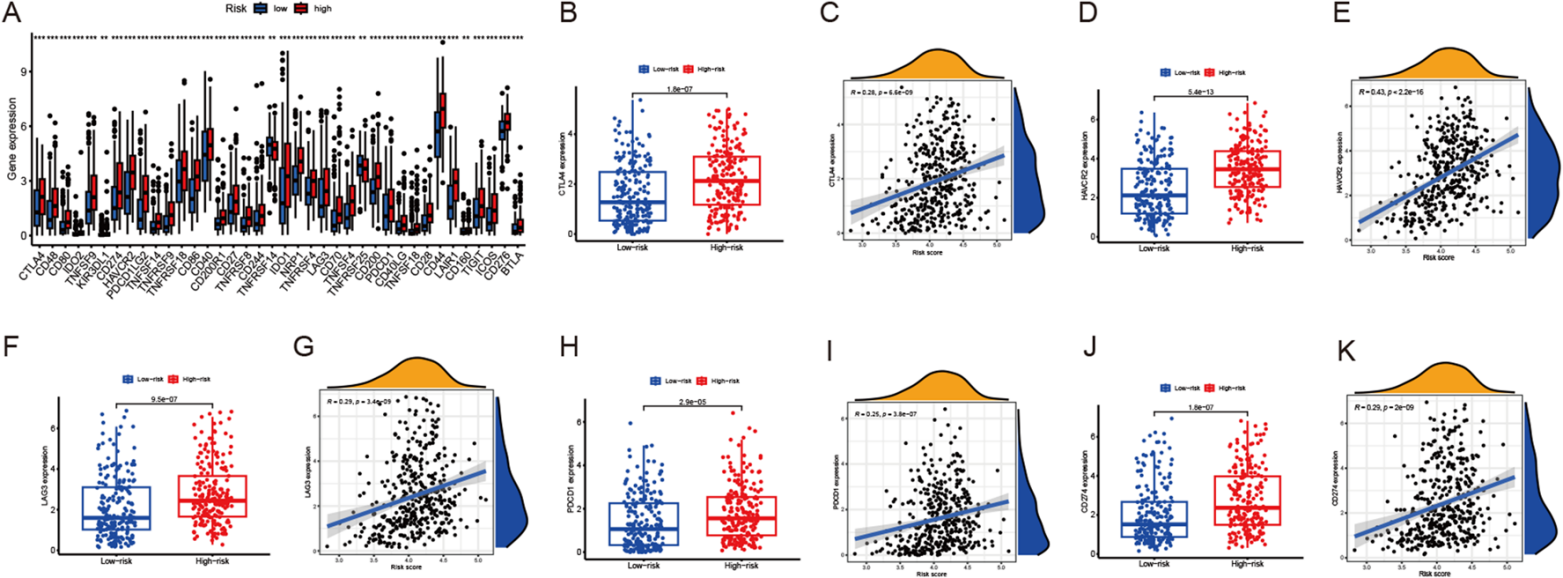
Correlation between tumor immune-checkpoint and risk score. **A** Comparison of the immune-checkpoint genes in the high- and low-risk groups. **B**–**K** Spearman correlation analysis between immune-checkpoint molecules and risk score

As for TME estimation, ESTIMATE algorithm was employed. Violin plot showed that stromal score, immune score and ESTIMATE score are significantly higher in high-risk group, indicating lower tumor purity (Fig. 6A). By CIBERSORT algorithm, we compared the difference of infiltration immune cells between high and low risk groups (Fig. 6B). The proportion of T cells CD8, T cells follicular helper, T cells regulatory (Tregs) was significantly higher in the low-risk group than in the high-risk group, while the proportion of T cells CD4 memory resting and Neutrophils were significantly higher in the high-risk group than in the low-risk group (Fig. 6C). To investigate their agreement with OS, survival curves were plotted. Except neutrophils, the results of coalition of immune cell with OS were in good agreement with immune cell infiltration results (Fig. 6D–G). Then we estimated the different immune functions between two groups. The heatmap indicated many immune-functions were higher enriched in high group, including T cell co-stimulation HLA and checkpoint (Fig. 6H). These results illustrated strong association between risk score model and tumor immunity. Furthermore, we identified gene expression of different checkpoint. We found that many checkpoint including CTLA4, CD274, also known as PD-L1, HAVCR4, LAG3 and PDCD1 were extremely higher expressed in high-risk group, indicating there might be different immunotherapy response between two groups (Fig. 7A). Spearman correlation analysis showed that CTLA4, HAVCR2, LAG3, PDCD1 and CD274 were significantly positively correlated with risk score (Fig. 7B–K). Generally, these genes were positively related to risk score.

### Prediction of immunotherapy response and potential drugs

**Fig. 8:**
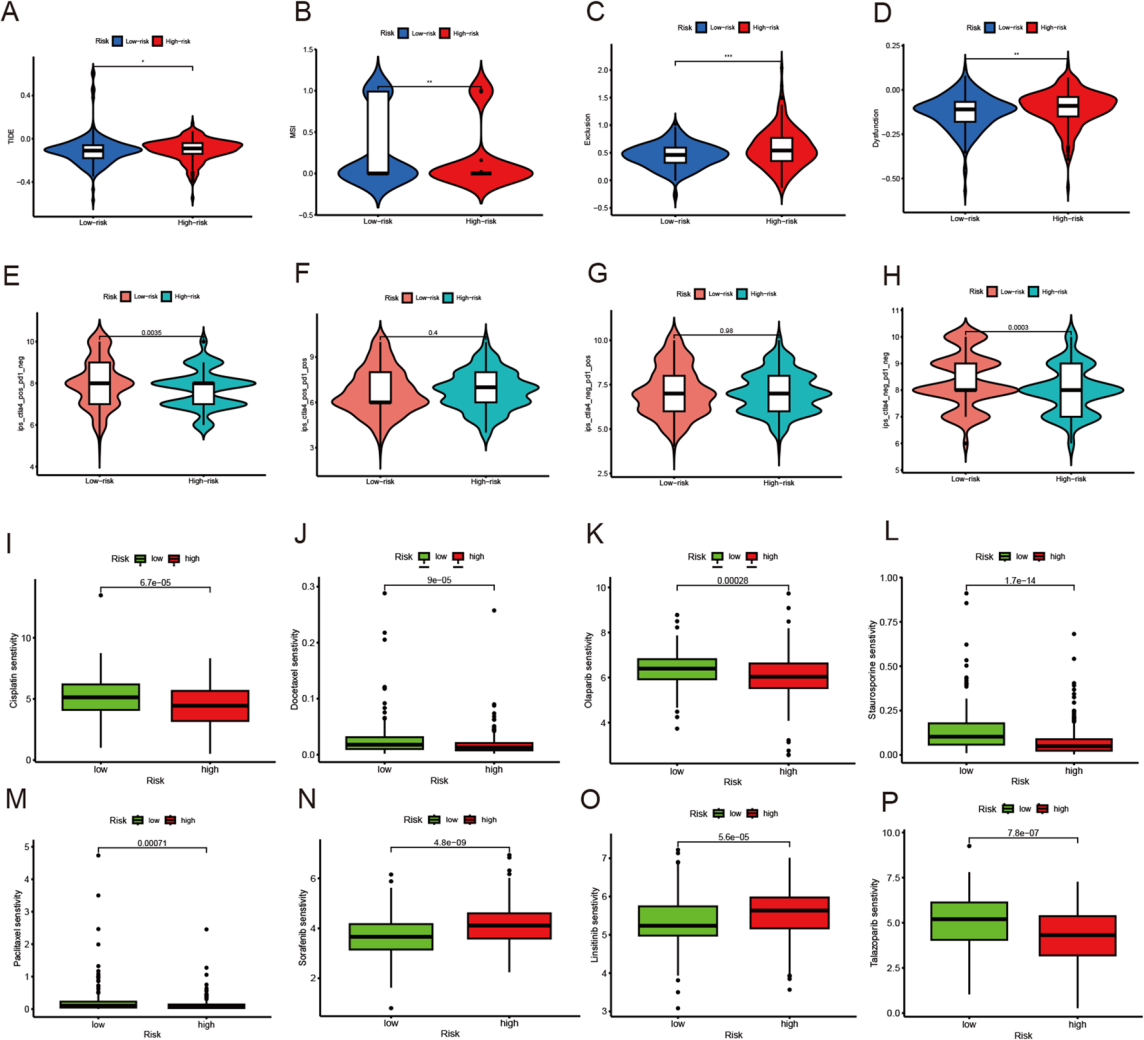
Assessment of response of high- and low-risk patients with BLCA to immunotherapy and potential drugs. **A**–**D** Immunotherapy response of BLCA patients in high- and low-risk groups. **E**–**H** The effect of immunotherapy between different subtypes. **I**–**P** The sensitivity of different drugs between the high- and low- risk groups

According to above results, we considered that our risk score model might play an important role in immunotherapy response prediction in BLCA patients. The TIDE algorithm showed that high-risk group had higher TIDE score, indicating the higher immune escape potential and poorer immunotherapy response of patients in the high-risk group (Fig. 8A–D). Therefore, we obtained immunotherapy file from TCIA database for further investigation. The violin plot demonstrated that the effect of immunotherapy was significantly improved in low-risk group when using anti-CTLA4 treatments (Fig. 8E–H). These results confirmed the strong association between risk score model and immunotherapy response in BLCA. Furthermore, we calculated the different drugs’ sensitivity of each patient based on GDSC database. Our risk score model had significant association with cisplatin, docetaxel, olaparib, staurosporine, paclitaxel, sorafenib, linsitinib and talazoparib (Fig. 8I–P). It might provide a novel prospective in BLCA treatment.

### Identifying TFRC as prognosis marker for BLCA

**Fig. 9:**
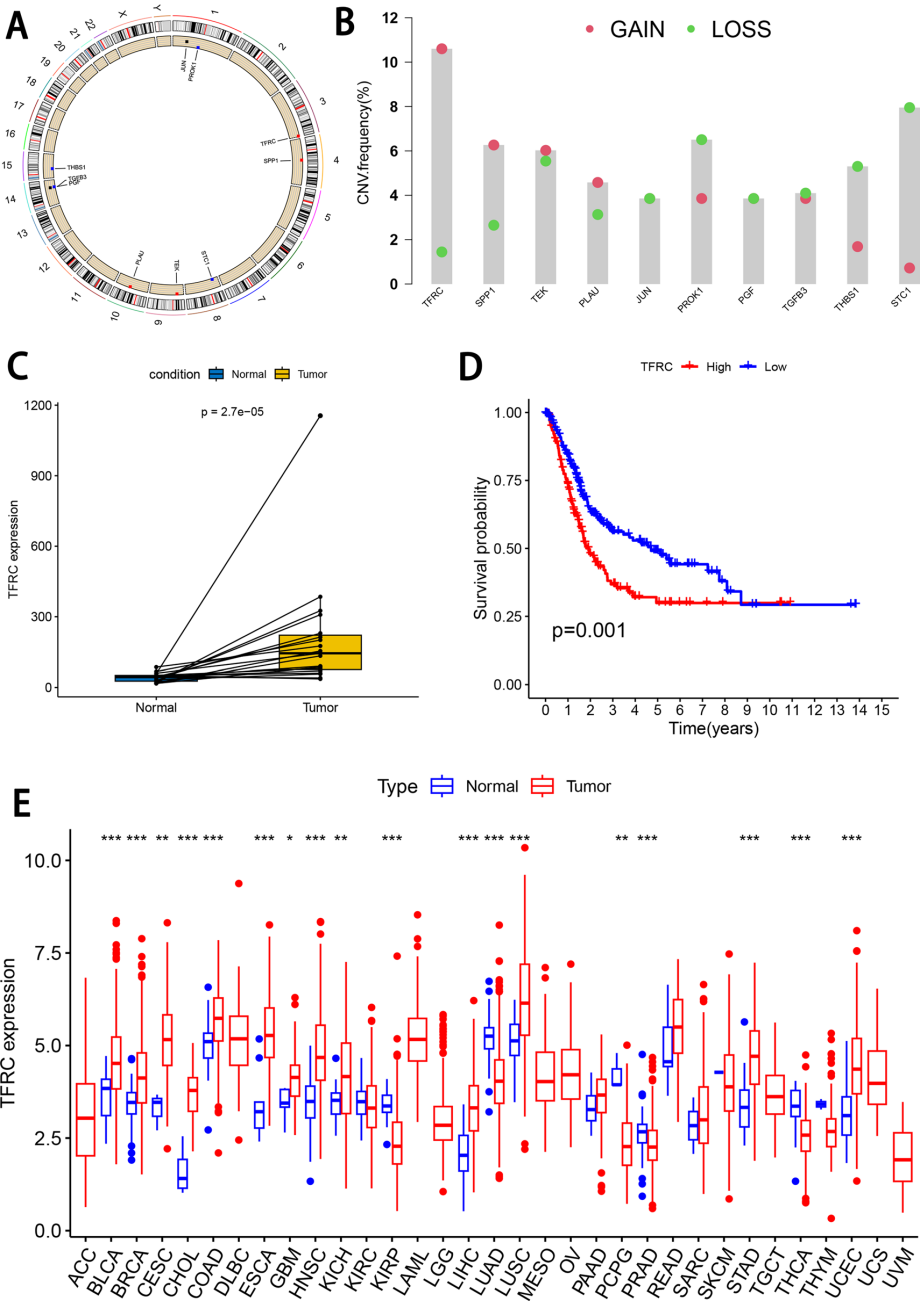
Identifying TFRC as prognosis marker for BLCA. **A** The position of TFRC in the chromosome. **B** The mutation frequency of the model genes. **C** TFRC expression levels of normal and tumor tissue in BLCA patients. **D** OS curves for BLCA patients with high and low TFRC expression. **E** The expression levels of TFRC in pan-cancer

The circus plot shows the position of the model gene in the chromosome (Fig. 9A). To identify hypoxia- and immune-related prognosis markers for BLCA, we analyzed the mutation frequency of the model genes via CNV, and the results showed that TFRC had the highest acquired mutation frequency (Fig. 9B). TFRC was significantly higher expressed in BLCA tumor samples compared with normal samples (Fig. 9C), and survival curves showed BLCA patients with low TFRC expression level had significantly longer OS compared with that with high TFRC expression level (Fig. 9D). The expression levels of TFRC in pan-carcer were also analyzed, and the box plot demonstrated differential expression of TFRC between normal and tumor tissue samples across multiple tumors (Fig. 9E). Spearman correlation analysis was conducted to demonstrate the relationship between TFRC expression and CTLA4 as well as PTCD1 (Additional file 2: Fig. S2A, B). This study discovered an association between TFRC and the immune microenvironment in patients with bladder cancer. This identification allows for the classification of bladder cancer patients into six immune subtypes, which aids in categorizing BLCA based on various immune response types (Additional file 2: Fig. S2C).

### Knockdown of TFRC inhibited BLCA cell proliferation, migration and invasion

**Fig. 10:**
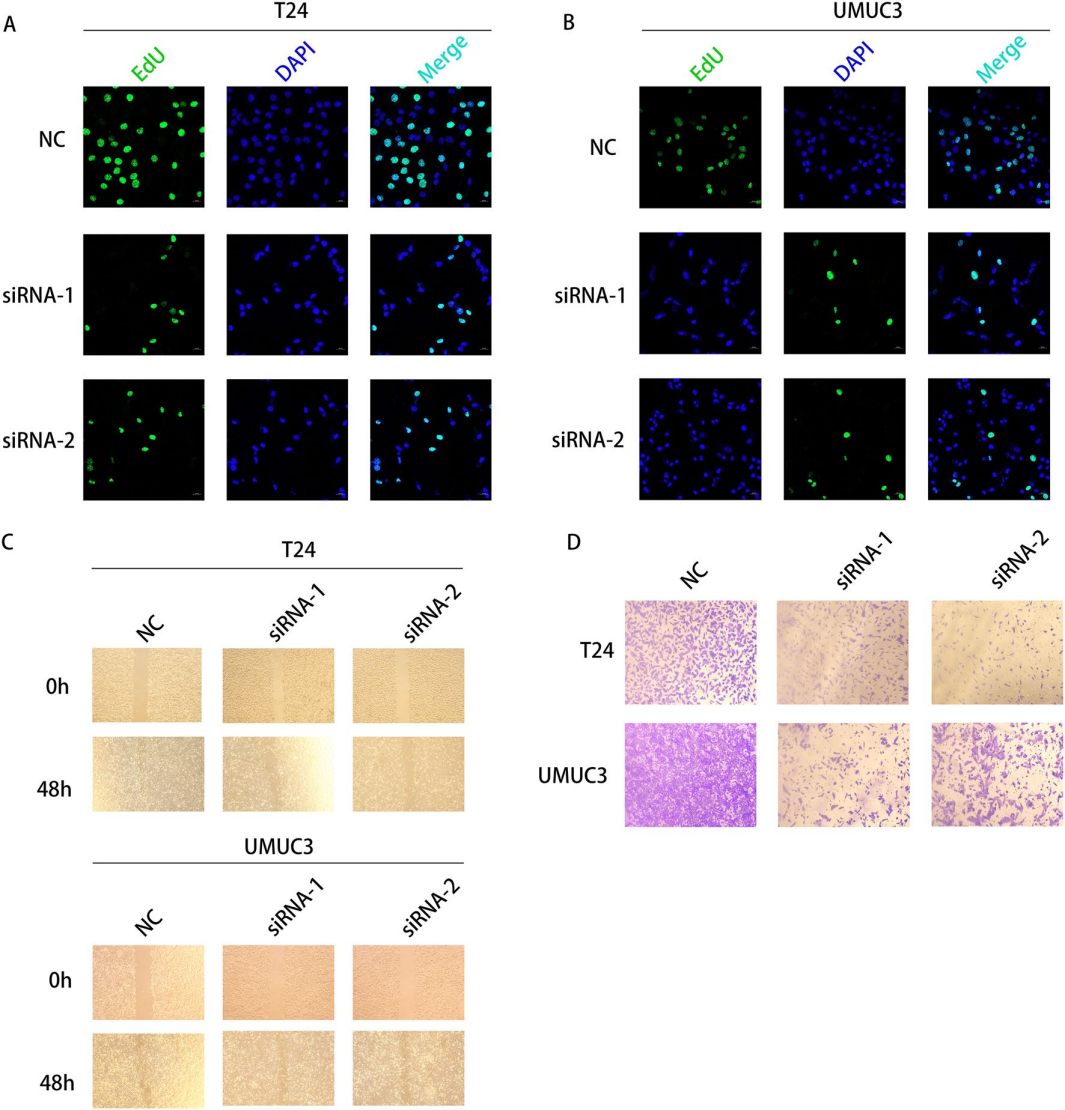
TFRC promotes the proliferation, migration, and invasion of BLCA cells. **A**, **B** The cell proliferation of control cells comparing to TFRC knockdown cells. **C** The cell migration of control cells compared to TFRC knockdown cells. **D** The invasion ability of control cells compared to TFRC knockdown cells

To validate the biological function of TFRC in BLCA, we knocked down TFRC using two siRNAs in T24 and UMUC3 cells. Edu staining results showed that knockdown of TFRC significantly reduced the proliferation of BLCA cells (Fig. 10A, B). The wound healing rate of T24 and UMUC3 cells was significantly inhibited by BLCA depletion, as indicated by the results of the wound-healing assay (Fig. 10C). The invasion ability of HepG2 and Hep3B cells was significantly inhibited in transwell assay following TFRC knockdown (Fig. 10D).

## Discussion

Bladder cancer is one of the most common malignancies worldwide, characterized by high morbidity and mortality [26]. Although researchers have made many attempts and explorations in the treatment of bladder cancer, the prognosis of patients with bladder cancer has not improved substantially due to high postoperative tumor recurrence rate [27]. Therefore, novel prognostic and outcome model for BLCA needs to be identified urgently.

Hypoxia plays a vital role in cancer cell survival as it can increase tumor cell proliferation and tumor cell transition, which causing malignant phenotype transition [28]. This might indicate that study tumor hypoxic environments could help the decision of BLCA treatment. Immunotherapy is an important option for BLCA patients. Thus immune cell infiltration and immune-related genes had gained much research [29, 30]. Although many prognostic models have constructed and predicted survival outcomes in BLCA based on hypoxia-related genes or immune-related genes, combing hypoxia- and immune-related genes is a novel method. When these two phenotypes were nested, the prognosis of patients could be more accurately evaluated and immunotherapy could be guided.

In this study, we identified 18 differentially expressed hypoxia and immune-related genes in BLCA patients through analysis of public databases. Using Cox and LASSO regression analysis, we developed a new prognostic model containing 8 genes (JUN, STC1, PROK1, TFRC, TGFB3, PLAU, PGF and SPP1) to predict overall survival in BLCA patients. Furthermore, Kaplan–Meier curves of disease-free survival (DFS) and overall survival (OS) showed that high-risk group had worse outcome. The consistent results were observed in test cohort, indicating this hypoxia- and immune-related gene risk score model can be used as a prognostic marker for BLCA. Furthermore, we confirmed this model had strong association with various clinical features. Besides, the impact of risk score model on the immune microenvironment, immune checkpoint, immunotherapy response, and anti-tumor drug sensitivity were also investigated.

JUN is a dimer and functions as a transcription factor and The heterodimers of JUN have a more stable and relatively stronger DNA-binding behavior [31, 32]. Research had confirmed the associated with the invasiveness of colorectal cancer cell [33]. STC1 is a hypoxia-induced molecular target which promotes the progression of different types of cancer, including gastric cancer [34], colorectal cancer [35], breast cancer [36] and bladder cancer [37, 38]. As a ligand of the PROKR2, PROK1 can induce biological changes by transducing important molecular signals [39]. PROK1 can influence the expression of EG-EGFR thus influence the cell proliferation and hematogenous metastasis [40–42]. TFRC is considered to be an important player in intracellular iron transport, which induces the proliferation and metastasis by up-regulating the expression level of AXIN2 [43]. Besides, many models containing TFRC had been confirmed to have associated with prognosis of bladder cancer [44–46]. In this study, we also found the role of TFRC in the proliferation of bladder cancer. We used siRNA to reduce TFRC expression in bladder cancer cells and found that TFRC knockdown inhibited tumor cell proliferation and reduced their invasive ability. AXIN2 might be one of the regulatory factors, but the specific mechanism needs to be further studied.

TGFB3, also known as transforming growth factor-B3, was associated with anti-PD-L1 monoclonal antibody treatment in urothelial carcinoma [47]. Inhibition of TGF-β signaling can conquer resistance to PD-1/PD-L1 blocking in cancer [48]. PLAU is involved in tumor cell migration and invasion [49], and previous study had confirmed the relationship between PLAU and OS in TCGA cohort [50]. PGF was also discovered to be associated with BLCA prognosis and OS [51, 52]. It has also been suggested that SPP1 was correlated with poor clinical outcomes and promote tumor progression by interacting with carcinogenic genes and facilitating immune cell infiltration [53]. High SPP1 expression levels were strongly connected with higher stage and grade in upper tract urothelial carcinoma [54].

After analyzing the different genes between subgroups, we found the PI3K-Akt pathway was activated in the high-risk group. PI3K-Akt pathway was a “star pathway” in the bladder which regulated autophagy, apoptosis and cancer progression. According to these results, we inferred that the willingness of high-risk groups to metastasize and muscle-invasive might be related to the PI3K-Akt pathway. Furthermore, we investigated the immune environment between subgroups. In hypoxic environments, macrophages synthesize chemokines and cancer cells attract regulatory T cells from the circulation and suppress the antitumor responses of other T cells [55]. In our study, we also found significantly immune cell infiltration including many types of T cells.

In this study, we demonstrated improved survival outcomes in the high TMB group, which is consistent with previous studies [56]. Groups with high TMB and lower risk scores had the best survival outcomes, whereas groups with low TMB and higher risk scores had the worst survival outcomes. Taken together, a combination of TMB and risk score could significantly improve the prediction of overall survival in bladder cancer. Other studies had found that in bladder cancer, high TMB status can increase the neoantigen burden and thus enhance the immunotherapeutic effects [56, 57]. Therefore, this risk score model might provide new insights into the underlying mechanism with high TMB.

Besides, tumor progression is usually affected by abnormal pathological conditions of tumor microenvironment, such as tumor-associated fibroblasts (CAFs), ECM deposition, vasodilation, and immune response suppression [58]. In this study, we employed ESTIMATE to evaluate components of tumor. The increase of matrix components and immune cell components indicates that the lower the purity of the tumor, the more likely the tumor metastasis and the worse the prognosis.

The immune response is considered to be the primary mechanism of action of the BCG, studies using factors reflecting the patient’s immune status to predict the effect of BCG treatment have been recently reported [59]. In a multicenter study of bladder carcinoma in situ, 70 years old was used as cut-off value, suggested that patients over 70 years old had an increased risk of recurrence and progression with a poor recurrence free survival. Elderly patients may not respond effectively to BCG treatment [60], indicating the treatment of these patients faces more challenges.

The immune system and tumor microenvironment are widely believed to be closely related to bladder cancer and play an important role in the development, maintenance and spread of bladder cancer, as well as the response to treatment [61]. With the development of genomics and the advent of immune checkpoint inhibitors, an increasing number of therapeutic targets are being identified [62]. For instance, modifications of oncogenes such as cyclin dependent kinase (CDK) and fibroblast growth factor receptor (FGFR3) can serve as predictive biomarkers for their respective inhibitor responses [63, 64]. Based on these results, we assume that patients in different subgroups might have different immune-checkpoint expression and response to immunotherapy. After investigation, we discovered many checkpoints including CTLA4, PD-L1, HAVCR4, LAG3 and PDCD1 had different expression, indicating different immune-response. As a novel cancer treatment option, immunotherapy gives hope to many bladder cancer patients. The correlation of immune infiltration with immunotherapy response in BLCA cases has also been reported [57]. We observed patients in the high-risk group had significant lower responses than those in the low-risk group for CTLA-4-positive or both negative. TIDE results indicated high-risk group was easier to have immune escape. Furthermore, we found that low-risk group was more sensitive to cisplatin, docetaxel, olaparib, staurosporine, paclitaxel and sorafenib. In conclusion, our risk model can assess the prognosis, immune status, immunotherapy response, and drug sensitivity of BLCA cases.

However, there are certain flaws in this study. First, this study was based on public database, thus the results may be skewed for different ethnic groups and regions. Second, the particular pathways might require further investigation with in vivo and in vitro experiments.

## Conclusion

This 8-genes risk score model was an accurate and reliable tool for predicting clinical outcomes, immunotherapy response, and anti-tumor drug sensitivity of BLCA patients, providing novel prospective for BLCA treatment. TFRC plays an important role in bladder cancer, which affects proliferation, invasion, and migration of BLCA. TFRC may be a very important target in the treatment of bladder cancer.

### Supplementary Information

**Additional file 1:** Heatmap of expression of 7394 genes in normal and tumor tissue samples.

**Additional file 2:** The relationship between TFRC and the immune microenvironment in patients with bladder cancer. A The relationship between TFRC expression and CTLA4. B The relationship between TFRC expression and PDLD1. C Different subtypes of immune response in patients with bladder cancer.

**Additional file 3:** Expression of 7394 genes in normal and tumor tissue samples.

**Additional file 4:** 18 differentially expressed genes related to hypoxia and immunity were identified.

**Additional file 5:** Eight genes that make up the prognostic model.

**Additional file 6:** 1547 differently expression genes between subgroups.

## Data Availability

Any data and R script in this study can be obtained from the corresponding author upon reasonable request. In this study, publicly available datasets were analyzed. These are available on The Cancer Genome Atlas (https://portal.gdc.cancer.gov/) and GEO (https://www.ncbi.nlm.nih.gov/).
