# Supplementary figures and images for: TFRC, associated with hypoxia and immune, is a prognostic factor and potential therapeutic target for bladder cancer
Source: Eur J Med Res. 2024 Feb 9;29:112. doi: 10.1186/s40001-024-01688-9 (PMC10854140; doi:10.1186/s40001-024-01688-9)

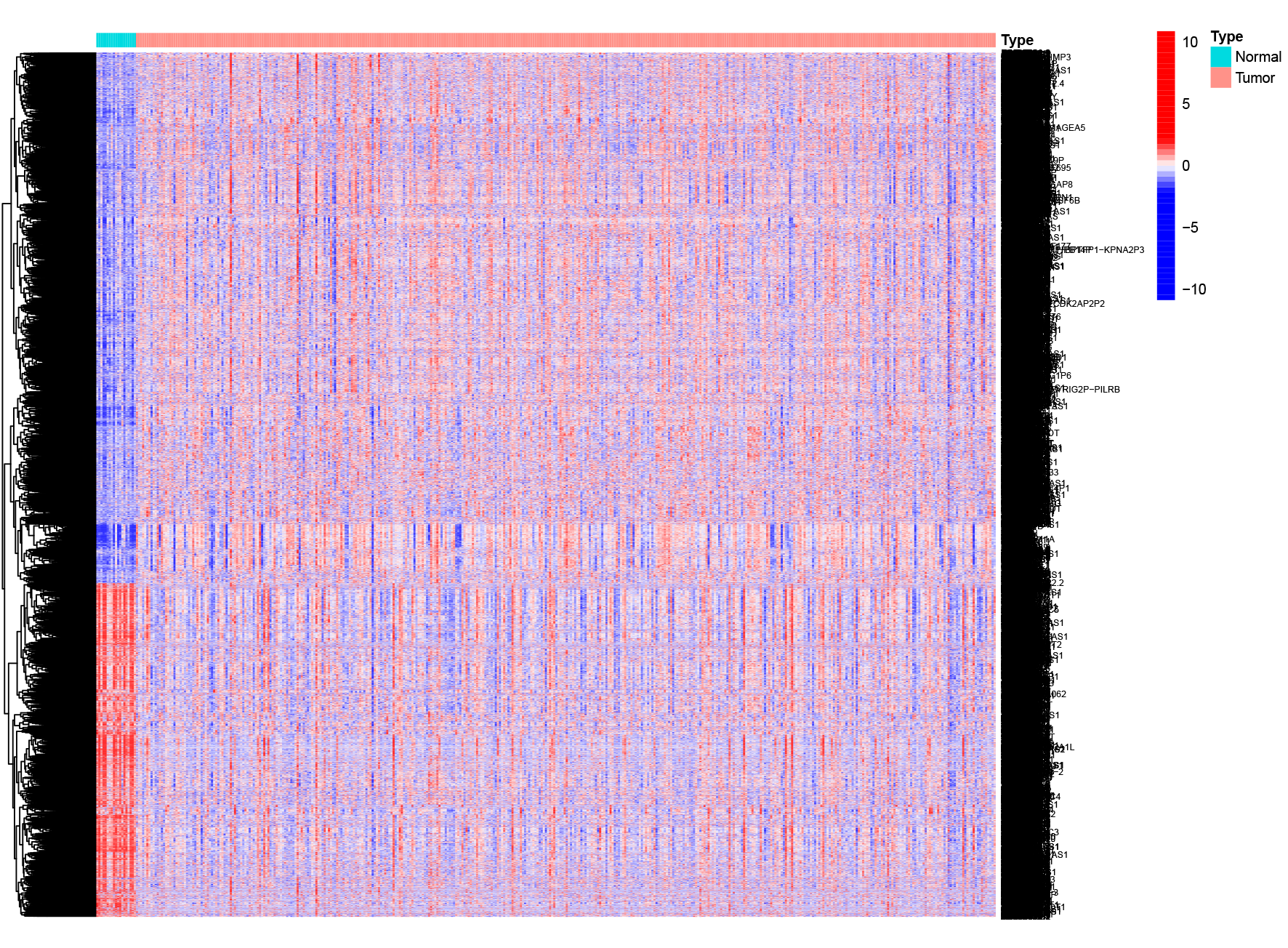

Supplement: Supplementary file 1 — Additional file 1: Heatmap of expression of 7394 genes in normal and tumor tissue samples. [file 40001_2024_1688_MOESM1_ESM.png]

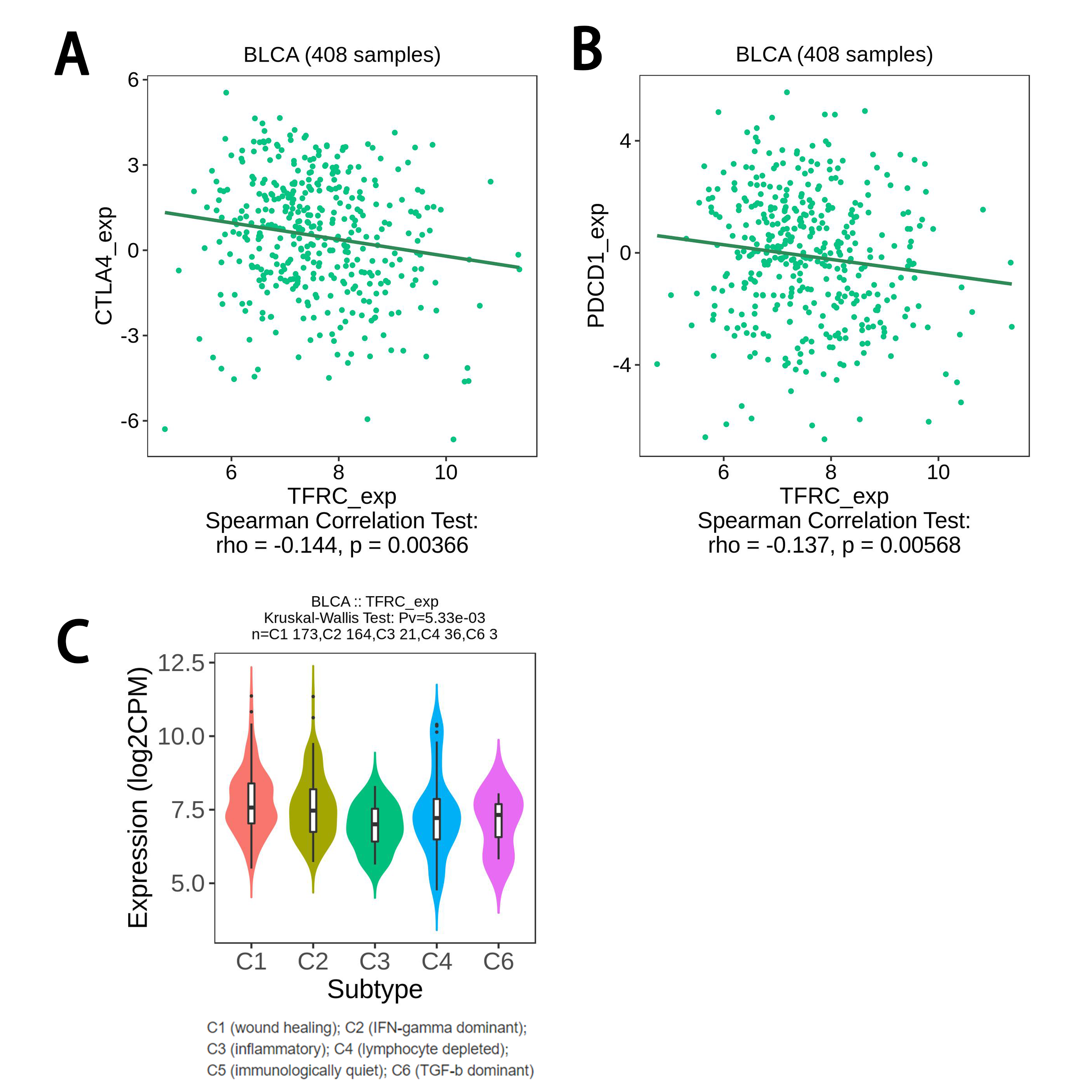

Supplement: Supplementary file 2 — Additional file 2: The relationship between TFRC and the immune microenvironment in patients with bladder cancer. A The relationship between TFRC expression and CTLA4. B The relationship between TFRC expression and PDLD1. C Different subtypes of immune response in patients with bladder cancer. [file 40001_2024_1688_MOESM2_ESM.png]
